# Decision-making ethics in regards to life-sustaining interventions: when physicians refer to what other patients decide

**DOI:** 10.1186/s12910-022-00828-2

**Published:** 2022-09-02

**Authors:** Anca-Cristina Sterie, Ralf J. Jox, Eve Rubli Truchard

**Affiliations:** 1 Chair of Geriatric Palliative Care, Palliative and Supportive Care Service and Service of Geriatric Medicine and Geriatric Rehabilitation, Lausanne University Hospital and University of Lausanne, Lausanne, Switzerland; 2 Service of Geriatric Medicine and Geriatric Rehabilitation, Lausanne University Hospital and University of Lausanne, Lausanne, Switzerland; 3 Service of Palliative and Supportive Care, Lausanne University Hospital and University of Lausanne, Lausanne, Switzerland; 4 Institute of Humanities in Medicine, Lausanne University Hospital and University of Lausanne, Lausanne, Switzerland

**Keywords:** Cardio-pulmonary resuscitation, DNAR, Doctor-patient communication, Conversation analysis, Nudges, Decision making, Social norms

## Abstract

**Background:**

Health decisions occur in a context with omnipresent social influences. Information concerning what other patients decide may present certain interventions as more desirable than others.

**Objectives:**

To explore how physicians refer to what other people decide in conversations about the relevancy of cardio-pulmonary resuscitation (CPR) or do-not-attempt-resuscitation orders (DNAR).

**Methods:**

We recorded forty-three physician–patient admission interviews taking place in a hospital in French-speaking Switzerland, during which CPR is discussed. Data was analysed with conversation analysis.

**Results:**

Reference to what other people decide in regards to CPR is used five times, through reported speech. The reference is generic, and employed as a resource to deal with trouble encountered with the patient’s preference, either because it is absent or potentially incompatible with the medical recommendation. In our data, it is a way for physicians to present decisional paths and to steer towards the relevancy of DNAR orders (“Patients tell us ‘no futile care’”). By calling out to a sense of membership, it builds towards the patient embracing norms that are associated with a desirable or relevant social group.

**Conclusions:**

Introducing DNAR decisions in terms of what other people opt for is a way for physicians to bring up the eventuality of allowing natural death in a less overt way. Formulating treatment choices in terms of what other people do has implications in terms of supporting autonomous and informed decision making, since it nudges patients towards conformity with what is presented as the most preferable choice on the basis of social norms.

**Supplementary Information:**

The online version contains supplementary material available at 10.1186/s12910-022-00828-2.

## Introduction

In the hospital context, anticipatory decisions about cardio-pulmonary resuscitation (CPR) are commonly made or reviewed already at the patient’s admission. The established standard for this decision-making process is the model of shared decision making, which is based on information sharing and bidirectional exchange between the patient and the physician in the spirit of a therapeutic partnership [1]. This model is aimed towards encouraging health professionals to support patients’ autonomous deliberation by enabling them to reflect about their preferences in accordance with values that matter most to them.

Research shows that CPR discussions are a frequent cause of ethical difficulty [2–4]. Our own results [5] show that explanations provided by hospital physicians about the CPR procedure are scarce, and, when existent, sketchy and simplistic, overlooking aspects related to prognosis or risk of adverse outcomes. Physicians seem to take for granted that CPR is understood by everyone and does not warrant explanation. The way that physicians ask patients about CPR does not always foster a well-informed exercise of patient autonomy [6].

According to the model of shared decision, physicians should have a “professional equipoise” attitude towards patients, by listing options that are reasonably available, including the option of not taking action, without having or displaying a preference about the treatment [7]. However, this ideal is hard to reach in practice. Studies describe that, even when physicians refrain from making overt recommendations, the way that options are presented functions as a recommendation for or against a particular course of action [8–10]. Communication research conducted on decision-making conversations can contribute to better understanding the challenges that patients and health professionals face in such contexts, for example by identifying resources employed to introduce, explain and negotiate decisions, as well as by reflecting on their ethical dimensions.

## Social comparison in healthcare communication and health decisions

Health decisions occur in a rich context in which social influences are omnipresent. One of the critical social factors impacting decision making is the tendency to compare oneself with others [11]. Indeed, research in social psychology has demonstrated that providing people with information about the frequency of an opinion (also referred to as “descriptive norms”) can influence behavior [12, 13]. As such, reference to what other people do, think or decide, qualifies as a nudge, in the sense that it can influence or alter people’s choices and behavior via “shallow cognitive processes” that operate on emotions [14, 15]. Other nudges frequently mentioned in the domain of health are use of incentives (receiving a more or less symbolic pay for engaging in certain activities), default rules (presetting options towards the preferred outcome, thus requiring more effort to opt out), framing that emphasizes salience and affect (describing something as novel, personally relevant, or through vivid descriptions), and priming (based on subconscious cues such as the order in which options are arranged or the associations used to offer them) [16]. The power that nudging strategies can have in changing human behavior has generated an ongoing debate on whether and under what conditions it is legitimate to encourage people to make “good choices” that improve their well-being when this encouragement undermines informed consent and patient autonomy [17].

One prominent strand of research on the influence of social comparison focuses on how social comparison can be a strategy for coping with illness. For example, Wood et al. discusse how, in 1985, many women with breast cancer lacked “comparison others” (relatives or friends who had lived through the same experience) and therefore tended to compare themselves to media figures, who disproportionately featured “supercopers” [18]. This upward comparison often made women feel inadequate. Whenever they could, women would rather use a downward comparison perspective, comparing themselves to people who were less fortunate, which enhanced their self-esteem.

Another strand of research of health communication research concerns how descriptive norms can influence a person’s perception of their vulnerability and their medical choices. Klein’s series of three studies showed that risk information containing social comparison has a greater impact on people’s emotions, intentions and behavior than personal risk information, and that favorable social comparison information has stronger influence than unfavorable ones [11]. French et al. [12] reported less difference between how personal and social comparison information impact emotional responses and perceptions of risk, though their consistent finding is that both types of comparative risk information had more impact on patient responses than information that didn’t include comparisons.

Here we present findings regarding how physicians use social comparison when talking with their patients about whether or not to opt for CPR, by referring to other persons’ decisions about CPR (for example, other patients). We describe how this reference is achieved in the conversation and explain what it accomplishes in interaction. Given the ethical ramifications of this topic, we conclude by discussing the implications of our findings in regard to decision making and clinical ethics.

## Participants and methods

### Participants

The study population concerned 43 patients transferred to the geriatric rehabilitation facility of a Swiss university hospital as well as the physicians who conducted admission interviews with them. Eligible patients had to have decision-making capacity for medical treatment, as determined by medical assessment (Author 2 or 3). All participating physicians were in their 1st–3rd year of medical practice after obtaining their medical qualification. Patient information was collected from the hospital chart, including whether code status (the type of treatment a patient would or would not receive in case of cardiac arrest) had been documented prior to the current admission (Table 1).

**Table 1:** Patient information

Age, mean in years	83.65
Documentation of a prior code status	
Yes	11 (25.6%)
No	25 (58.1%)
Not available / unclear	7 (16.3%)

In the year in which started the study, hospital statistics for the service in which we recorded showed that for 43% of the admitted patients CPR was documented as relevant and for 56% not.

### Data collection

Our dataset comprises 43 recorded and transcribed CPR discussions occurring during admission interviews between 43 patients and 17 physicians. Data was collected over a 10-month period (April 2017–January 2018).

Physicians who routinely conduct admission interviews were asked for their permission to audio-record these interviews. They were instructed on how to use the recording device and to switch it on when starting the admission interview. Informed consent to audio-record the patients’ admission interviews was obtained 24–48 h prior to their transfer to the rehabilitation facility (by Author 1). The study was conducted with the approval of the Vaud Ethics Committee.

### Data analysis

The recordings were transcribed using Jeffersonian conventions [19] (see Additional file 1: Appendix 1) and translated from French for the purposes of this article. In the transcripts that we show here, physician’s turns are marked by the abbreviation (PHY) and patient’s by (PAT). The arrows (→) signal when the physician employs social comparison in the conversation and refers to what other patients decide in regard to CPR. Original French transcripts can be found in Additional file 2: Appendix 2 (Transcript 1) and Additional file 3: Appendix 3 (Transcript 2), which contains, when relevant, an additional line of transcription (in italics) when French words are translated into English, but the word order is left as the original so that the reader can easily see where transition relevance points, inbreaths, overlaps and word stretching occurs in relation to the words [20].

We conducted a conversation analysis (CA) of the data. CA consists of a finely-grained analysis of recorded data, focusing on how participants interact in the conversation in order to accomplish ordinary as well as interactionally challenging tasks [21, 22]. CA is concerned with how real-time talk is produced by using observational techniques that reveal the linguistic details of the conversation. Compared to other qualitative approaches, the type of data with which CA works (recordings of interactions happening spontaneously and independently of the research objective) allows a more accurate depiction of the intricacies of conversation. While CA is an inductive and exploratory approach, its use is regulated by a well-defined and step-wise process [23]. The analysis begins with observation (listening and watching) of the data. The goal is for an "unmotivated" [24] analysis of the interaction, bearing no prejudice based on expectations. This analysis is done as an “initial noticing”, in order to identify details of talk that are interesting from a research point of view but also recurrent throughout the data. Secondly, the researcher starts an exhaustive search throughout all the database of instances in which the phenomenon in question is produced, and gathers them in a data set. Sequences of talk in which the phenomenon is identified are transcribed according to a CA convention system which takes into account aspects of speech delivery (intonations, loudness, emphasis), relationships between part of talk (overlapping, silences) [19] and, when video data is available, representation of activities parallel to talk (eye gaze, laughing) [25]. Third, the essential part of the analysis involves describing the phenomena in terms of sequential location (where in the conversation it appears, what generates it, what it generates), form and function. Analysis benefits from regular inputs from fellow analysts, during data sessions, that ensure a shared understanding of the data. Given this approach, findings obtained with CA can be meaningful to beneficiaries and stakeholders, which supports comparative work as well as the development of communication trainings.

Over the past years, CA has been extensively used for the study of medical consultations [26–28]. CA applied to medical interaction focuses on identifying the social actions and activities that health professionals, patients and relatives accomplish during a medical encounter, the practical problems that they may face during these interactions, and what interactional resources they use in order to accomplish their goals. Extensive research has been dedicated to the context of primary care settings, looking at treatment recommendations [29, 30] as well as the delivery and receipt of diagnoses [31, 32]. In recent studies, interest is also drawn by sub-specialties linked to managing end-of-life that require particular sensitivity and revolve around decision-making, such as palliative care, oncology or neurology [28]. For example, Pino et al. [33] report how palliative doctors engage patients and their companions in end-of-life discussions. They investigate a particular practice, that of “open elaboration solicitations”. In this practice, doctors don’t overtly ask about end-of-life matters and avoid assuming that patients have any such concerns, thus giving precedence to patients volunteering end-of-life considerations on their own.

The practice we describe here (reference to other people’s decisions) appears in 14% of the data (five conversations). We analysed each conversation individually and comparatively in terms of “why that now”, the pervasive scientific and methodologic question that guides CA analysis [34]: what brings about the practice under scrutiny, how it is accomplished, how participants orient to it, and what its implications are in terms of patient involvement in decision-making, especially in regard to autonomy. Due to space restrictions, we present here two examples of conversation segments in which reference to other people’s decision is used. We selected these two excerpts based on them being the best exemplifiers of common and different aspects in regard to how the phenomenon appears in our data and to what purpose. For ease of reading, we use conventional orthography when citing excerpts in running text.

## Findings

Below (Table 2) we present excerpts from five conversations in which physicians refer to other patients’ decisions (in italics).

**Table 2:** Quotes containing physicians’ reference to other patients’ decisions about CPR

Occurrences	Quote (Original language—French)	Quote (Translated-English)
Conversation 1 (Fig. 1)	“Qu’est-ce que vous souhaitez qu’on fasse si jamais votre cœur s’arrête ? (…) Je pose cette question simplement parce *qu’il y a des gens pour qui c’est très clair que, ils disent non mais écoutez si ça s’arrête, moi j’ai bien vécu, je veux plus rien faire, et il y en a d’autres qui, voilà, ils disent ‘non, mais moi c’est quand même important qu’on fasse quelque chose.*”	“What would you wish that we do if your heart stops? (…) I ask this question simply because *there are people for whom it’s very clear that they say ‘no but listen if it [the heart] stops, myself I’ve lived well, I don’t want to do anything else. And there are others who, well, they say no, but myse*lf it’s actually important that we do something.”
Conversation 2 (Fig. 2)	“On ne peut pas prévoir les complications quand on essaie d’intervenir comme ça (…) c’est ce qu’on appelle, *souvent les patients nous disent ‘pas d’acharnement*”	“We cannot forsee the complications when we try to intervene like this (…) *It’s what we call, often the patients they tell us no futile care.*”
Conversation 3	“Alors, l’acharnement on ne le fait pas. *C’est tout le monde dit pas d’acharnement*, l’acharnement c’est aller faire trop de choses là où il n’y a pas à faire”	“So, we don’t do futile care. *Everyone says no futile care*, futility it’s to do too many things where there’s no need to do.”
Conversation 4	“Si on fait une réanimation, donc un massage cardiaque, on peut pas savoir comment ça va se passer, d’accord ? C’est selon le souhait du patient. *Si le patient, pendant qu’il pouvait parler, il nous avait dit qu’il voulait une réanimation*, on fait la réanimation, mais après il peut avoir des séquelles. Parce que pendant que le cœur il s’arrête, il n’y a pas de sang qui arrive dans le cerveau”	“If we do a resuscitation, so a cardiac massage, we cannot know how it will go, all right? It’s according to the patient’s wish. *If the patient, while he could still talk, had said that he had wanted a resuscitation*, we would do the resuscitation, but afterwards there may be secondary effects. Because while the heart stops, there is no blood flowing to the brain”
Conversation 5	“C’est une question qu’on pose à tout le monde, mais pour vous, jusqu’où est-ce qu’il faut aller en termes de soins ? Est-ce que par exemple si vous avez le cœur qui s’arrête parce qu’on se rend compte qu’il y a quelque chose de grave, à priori ce n’est pas le cas, mais est-ce que vous voulez qu’on fasse tout, tout, tout? (…) *Ou bien est-ce que vous êtes plus du style à dire on fait le maximum de ce qu’on peut, mais je ne veux pas qu’on réanime le cœur et je ne veux pas qu’on m’intube* ?”	“It’s a question that we ask everybody, but for you, to what extent should we provide care? For example if your heart stops because we realize that there’s something serious, presumably it’s not the case, but would you want that we do everything, everything, everything? (…) Or *are you the type who says we do the maximum of what we can, but I don’t want to resuscitate the heart and I don’t want to be intubated*”

Throughout the data, physicians might refer to other patients on multiple occasions, such as when they talk about the fact that CPR is discussed with all patients (“It’s a question we ask everybody”) [6]. Here, we are specifically interested in the instance in which physicians refer to other patients’ decisions in regard with CPR. The reference concerns a generic patient, presented as a collective: “some patients” (Conversation 1), “the patients” (Conversation 2), “everyone” (Conversation 3), or as a hypothetical patient: “the patient” (Conversation 4), “the type” (Conversation 5). The reference never addresses an actual patient.

**Fig. 1:**
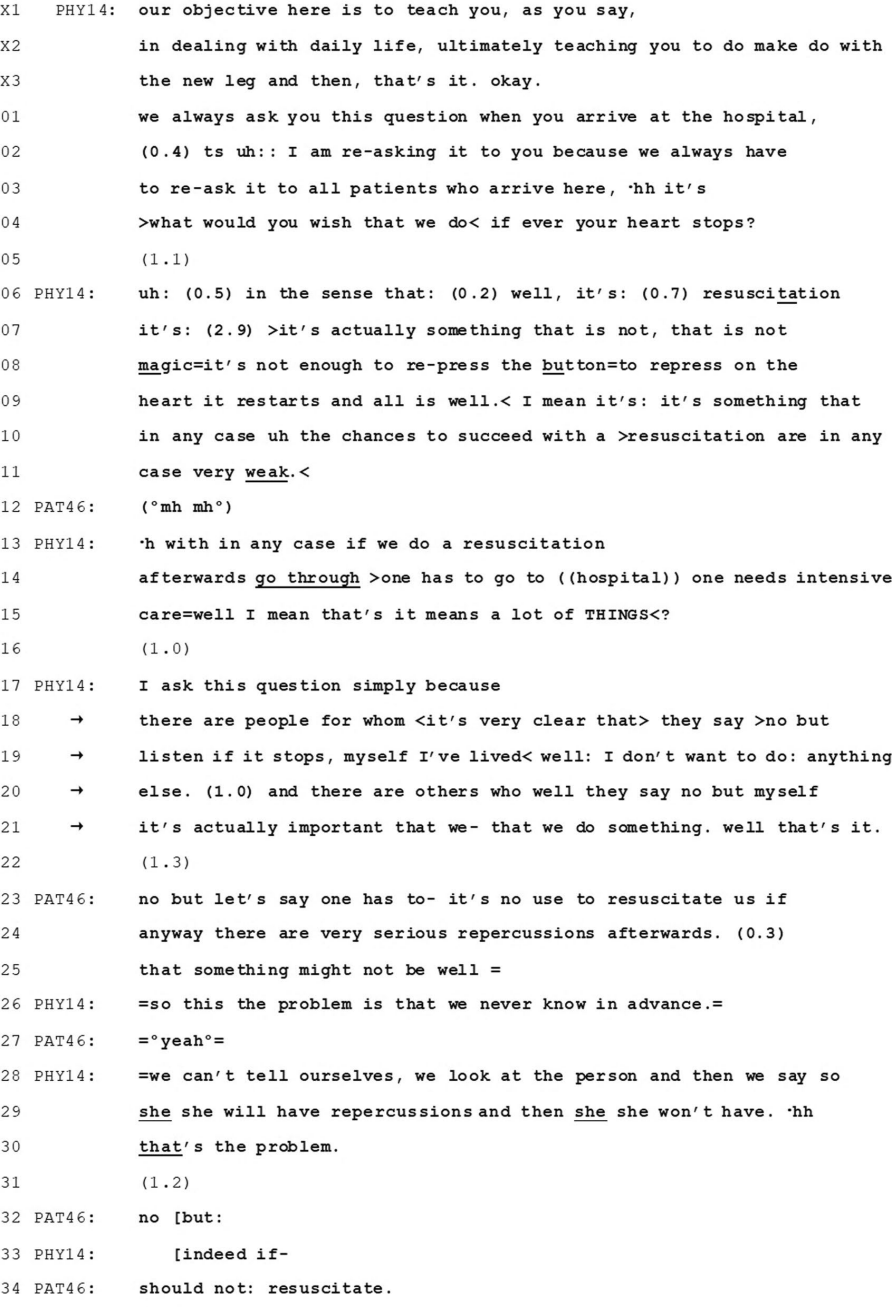
Transcript excerpt from conversation 35 (Physician #14: PHY14; Patient #46: PAT46)

**Fig. 2:**
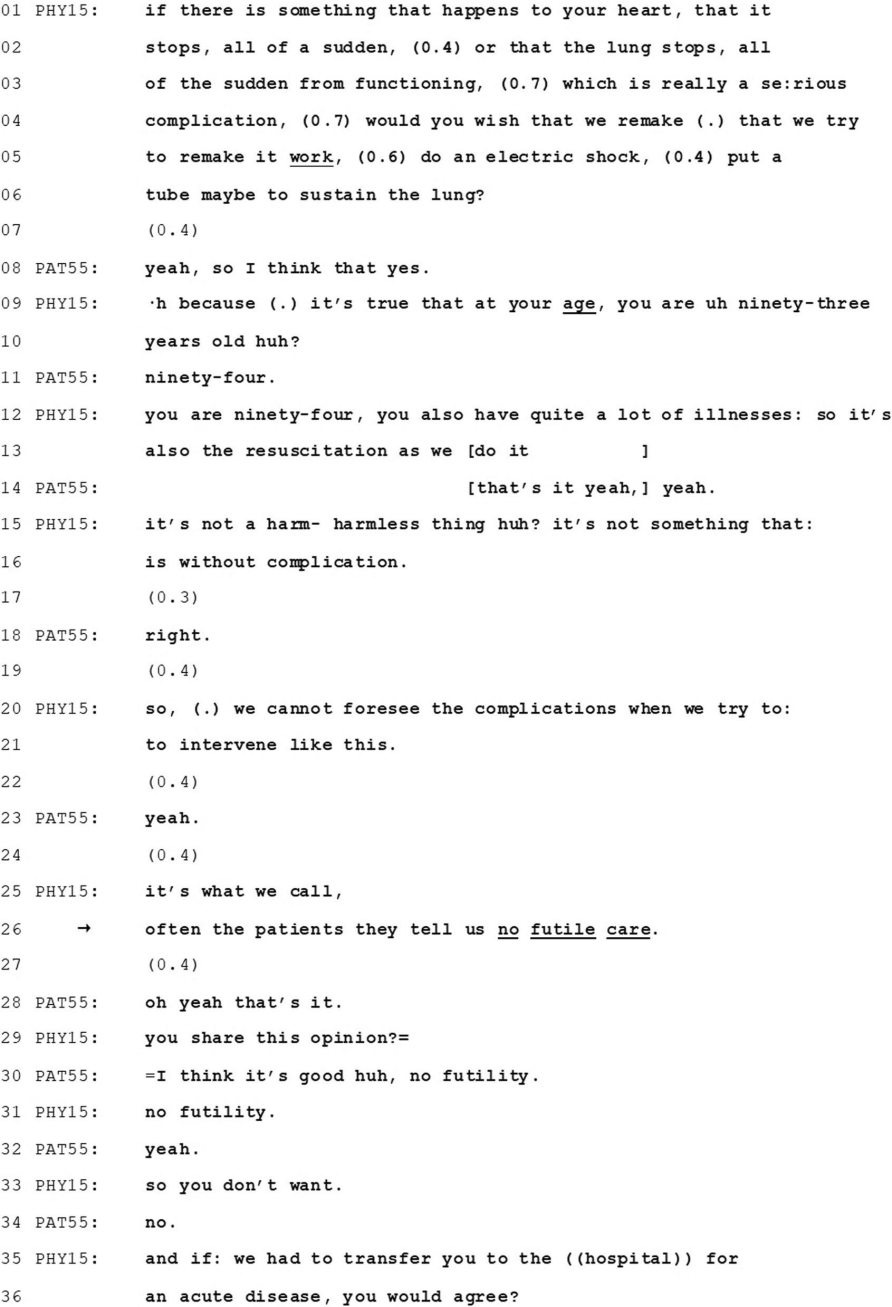
Transcript excerpt from conversation 44 (Physician #15: PHY15; Patient #55: PAT55)

The reference is used after the patient has already been given a chance to state their preference regarding CPR (for example, after addressing them a specific question about their preferences in terms of what should be done in case of cardiac arrest). As we will show, it is a resource employed to deal with a potential “trouble” with the patient’s response. Furthermore, this reference is associated with talk about orders for do-not-attempt-resuscitation (DNAR) being the most relevant decision in the given case. We exemplify these findings below through excerpts (in Figs. 1 and 2).

In Fig. 1 we present a transcript excerpt from the admission interview between a resident physician (PHY14) and a 76-year-old female patient (PAT46), admitted for rehabilitation after hip prosthesis surgery. Her previous code status was undefined. CPR is introduced at minute 10 of the 48-min-long admission interview. At this point, the conversation transitions from the physician stating the therapeutic objective during the patients’ stay at the facility (lines X1-X3) to talk about CPR. Reference to other patients’ decisions concerns both CPR and DNAR, and is made by the physician, in an effort to obtain a response (and decision) from the patient.

The physician introduces the topic of CPR as something routine (“we always ask”) and treats the matter as having already been discussed (“re-ask” in line 03), thus also implying and expecting a certain familiarity of the patient with the topic. Nevertheless, the patient’s chart at the moment of the discussion contained no information about whether CPR had been discussed recently or not. This type of introduction to a new topic on the agenda of the medical interview, scattered with anticipatory accounts, shows that the matter is treated as being sensitive and projects the topic as being potentially problematic [35–37].

The physician elicits the patient’s preference through a patient view elicitor (“what would you wish that we do”, line 04) [38]. Compared to other patient view elicitors described in CA literature about decision making, its particularity is that it is accomplished through an open question that, while conveying the idea that several options are available, doesn’t specify or list them. Thus it treats the patient as being familiar with these options, and gives the patient full decisional power over naming and selecting the most relevant one [6].

A response from the patient would have been relevant at this point, but is not forthcoming (line 05). In CA literature it was shown that such absences or significant delays in responding to a question are often associated to activities such as disagreement, disconfirmation and rejection of what was initiated in the sequence just before, which are “dispreferred” in interaction [39]. This implies that the noticeable silence of more than 1 s in line 05, even though void in terms of actual content, signals that the patient treats the question as unanswerable at this point.

In what follows, the physician employs different strategies to pursue a response from the patient [40], all based on providing the patient with more clear options among which she can chose. Initially (lines 06–15), he explains what CPR consists of. The cautious yet rapid description, with multiple repetitions and breaks, is typical of how people might raise “delicate’ problems” [41]. Together with the disparaging description of CPR (“not magic”, “not sufficient”, “weak”) this way of talking about CPR presents it as least preferred option, nudging in favor of electing to not attempt CPR. In the midst of this explanation, the patient is presented a first opportunity to intervene at turn completion in line 09 (after the explanation of why CPR is not magic). A new opportunity is offered upon establishing that the success rate of CPR is weak (lines 10–11), and this time the patient acquiesces (line 12), yet does not treat the information provided so far as sufficient or warranting a decision from her part.

More time for a response from the patient is offered in line 16, though she again remains silent. Again in pursuit of a response, the physician lays out decisional options by referring to what other “people” decide (lines 17–21): foregoing any action-taking in case of cardiac arrest (“I don’t want to do anything else”, lines 19–20) and wanting to undergo such interventions (“myself it’s actually important that we- that we do something”, lines 21–22). Each option is detailed and presented by use of direct reported speech. An opportunity for patient response is offered after presenting the first option (a silence of 1 s, in line 20), after which the physician continues with presenting the second option.

Presenting possible options underlines that there is choice and highlights the patient’s autonomy and involvement in choosing [9, 42–44]. However, the way that information is framed shows a potential preference towards one. In addition to the fact that in lines 06–13 CPR was described in very critical terms projecting its undesirability, in lines 16–18 DNAR is presented embedded into a positive evaluation of the quality of one’s prior life (“I’ve lived well”). Given that it is preferable to belong to the category of people who have “lived well”, this also sets DNAR as being the most preferred decision, while nonetheless leaving it up to the patient to affirm it.

In her response, the patient opts for not receiving CPR, though this decision emerges over several turns at talk. Initially (lines 21–23), she formulates an assessment that is as generic as the physician’s, referring to the futility of CPR on the basis of risk of suffering of repercussions (something that was not introduced by the physician). Through it, she responds quid pro quo to the physician’s mention of what other patients’ opt for, talking of herself in terms of collectivity (“no use to resuscitate us”, line 21). After the physician addresses the factor of suffering from adverse effects after a CPR (lines 24–26), the patient finally provides the decision (“no but should not resuscitate, line 30 and 32). Through the way it is designed, we see that this response turn is oriented towards refusing CPR (“no”).

The particularity of the excerpt resides in the fact that the physician frames options in terms of decisions that other people make, and employs this reference to present both potential choices. By presenting himself as someone who discusses CPR with all patients and in the context of his regular job as physician (“we have to re-ask”), the physician also presents himself as knowledgeable and able to share an overview over the options available, by whom they tend to be chosen and for what reasons. This allows him to assume the role of someone reporting what he has experienced and, by this means only, to formulate the possibility of allowing natural death. The reference is also decision-implicative. While the physician doesn’t overtly recommend this option to the present patient, the social comparison makes clearer what the available options are, and also introduces a subtle hint towards which option might be more desirable (DNAR), thereby slightly nudging the patient towards it. For the patient, making a decision becomes an issue of identifying with a category of people: those who “lived well” or those who want for “something” to be done (which she clearly makes, when she says that “it’s no use to resuscitate us”). Making this a membership choice removes the focus from the individual and the relevancy of personal health status and prognosis, to orient it towards some kind of pre-set choices available for certain categories of people. Nevertheless, the strength of this nudge is diminished by the fact that two options are presented and both framed as choices.

It is also worth nohing that the physician employs the reference to other patients’ choices as a pursuit of a response, i.e. when a response or decision could not be obtained by other means from the patient. Indeed in this conversation, several opportunities were given to the patient to formulate a response, among which the most noticeable were the formulation of a request (the open patient view elicitor) and the depreciative description of CPR. Use of social comparison is therefore a resource for clarifying the options and the activity at work (asking for patients to deliberate). The detailed work that is involved in introducing and formulating this reference, the delay with which the option of DNAR is formulated, as well as the use of a subtle nudge towards the relevancy of a DNAR order, show that actually wording-out options or recommendations towards foregoing CPR orders might be a challenge for physicians.

In Fig. 2, we present a transcript excerpt from another admission interview, in which the resident physician (PHY15, different from the one in Fig. 1) also refers to what other patients decide in regard to CPR. The patient (PAT55) is a 94 year-old female and is admitted for rehabilitation following epileptic seizures and pneumonia. Prior to admission for rehabilitation, her code status had been “no CPR”. At admission for rehabilitation, CPR is addressed at the beginning of the history taking, after inquiry into living relations. The physician refers to other patients refusing what is described as “futile care”; this happens after the patient has indicated a desire to be resuscitated.

The physician uses an explicit question which sets doing CPR as potential and sole course of action (“Would you wish that we remake, that we try to remake it [the heart] work, do an electric shock, put a tube maybe to sustain the lung?”, lines 04–06). While actual CPR terminology is not used, the course of action is described as a series of steps (shocking the heart and intubating the patient).

Initially, the patient claims a preference in favor of CPR (line 08). This is achieved through an initial agreement (“yeah”), followed by an epistemic marker (“I think so”) that downgrades the strength of this acquiescence [45, 46]. This might be attributable to the fact that the patient is conscious of choosing something that as presented is dispreferred terms by the physician (due to the choice of words that depict invasive activities—electric shocks and intubation).

The physician doesn’t treat the patient’s choice of CPR as an adequate answer, since he pursues a different decision, as demonstrated in the rest of the extract, [40]. Firstly, he provides information about personal prognosis, that the patient’s age and health might trigger “complications” in the CPR process (lines 09–16, and lines 20–21). While the patient provides acknowledgments of understanding related with such personal prognosis (line 14, 18 and 23), she does not review her decision.

Still in pursuit of another type of decision, the physician refers to “futile care” as something that patients generally decide against (“it’s what we call, often the patients they tell us no futile care”, line 25–26). This allows to associate “futility” to the measures presented in the beginning of the conversation (“make an electric shock”, “put a tube”) and to which the patient initially agreed. The decision is attributed a specific group: “the patients” (line 26). The reference is actually repaired [47], being initially projected as something physicians (“we”) might decide and then corrected to “the patients”. This allows to identify the decision against futile care as relevant based on the patient’s belonging to a homogenous group and on its ownership by that group (“they tell us”), and, based on this, projects it as most preferable option in this case. This also makes it difficult for the patient to dissociate from this group and from the decision that is linked to it.

As in the excerpt presented in Fig. 1, the physician is using direct reported speech to bring other people’s experiences to the table, which not only presents options but also normalizes specific choices. Quoting what other people decide is achieved by referring to physician’s experience, which makes it all the more difficult for the patient to challenge the option that is framed in preferential terms. Compared to the excerpt in Fig. 1, we can note that while the first physician used the references to other patients as a way to introduce both decisions in favor of and against CPR, here the second physician uses it to introduce only one alternative (against CPR). The weight of the nudge is therefore greater in this second example.

The patient recognizes the formulation of “futile care” (“oh yes”) and confirms this line of decision (line 28). In the following lines, the physician confirms three times whether the patient maintains the decision (though this doesn’t allow for any elaboration that would show what the patient makes of it), and finally goes on with the interview (line 35–36).

## Discussion

We will first discuss how reference to other patients’ decisions is used as interactional resource by physicians when talking about resuscitation preferences. We will then discuss this finding from an ethical point of view.

### Referring to other patients’ decisions as interactional resource in decision-making conversations

The practice of referring to other patients’ decisions has also been identified in other studies on how patients and physicians discuss CPR, as a way to provide a recommendation to not undergo the procedure [48], though never analysed in a detailed way. We review here the principal features and functions of this reference as encountered in our data.

First of all, referring to other patients’ decisions is a resource for presenting and describing options of choice in an explicit way. It is not the only resource to do so, as physicians also use patient view elicitors that clearly mention specific options (“would you wish that we try to re-make [the heart] work”), as well as descriptions of a particular option (as in Excerpt 1) or of the patients’ prognostics in relation to certain options (as in Excerpt 2), which also make decisions relevant. In our data, reference to other patients’ decisions is achieved by referring to a generic person or group of persons, never to a specific individual. This allows to identify options and enhances the validity of the choices by identifying them as potential decisional paths. The reference is formulated through the use of reported speech, which establishes a context of evidence. The fact of knowing what other patients opt for is confirmed by presenting it as coming from direct experience and as originating from a reliable authoritative source (since allegedly a patient or patients talked about their preferences) [49]. In the excerpts that we presented, physicians present both types of evidence, as they assert the direct experience of an authoritative source (the patient). The claim of reliable evidence is heightened by the use of direct reported speech, since it allegedly presents someone else’s talk in objective terms [50]. However, when the physician presents a therapeutic option in a context of evidence such as the one established when referring to what other patients opt for, it sets constraints that are difficult to escape for the patient. In this case, the decisions laid out are presented as belonging to a category of individuals to which the patients in these conversations belong objectively (“patients”) or which are desirable (people who have “lived well”).

Second, referring to other patients’ decisions is a resource for steering, more or less strongly, towards a particular option—in this data, DNAR. Use of the reference to other patients in this particular context of decision making allows for an interesting reflection on epistemics [51] and deontics [52], which is instrumental for understanding what exactly is accomplished by its use in interaction. When providing information about other people or patients, physicians claim an epistemic status [51] that is superior to the patient they have in front of them at the moment, since they can refer to the general patient cohort (while the patient in front of them only has access to their own preferences). The valence of what is conveyed through this reference is heightened by the role that the physician plays in the interaction, the rights and obligations in requesting and performing certain actions—what is called the “deontic status” [52]. This epistemic positioning and deontic status are important aspects for determining how what the physician says can be understood by the patient—in this situation, not as a mere information but as a recommendation of selecting a particular option because more adapted than any other. The nudging valence of this reference depends from case to case. In the conversation presented in Excerpt 1, it is only slight, since two options are introduced and presented as valid choices, thus leaving more freedom for patient’s deliberation. The only way in which DNAR is presented as preferable is by attributing it to a group of people who have “lived well” and by referring to it first. In Excerpt 2, however, the reference achieves a greater nudging function, since only the option of DNAR is described and attributed to “patients” in general, without providing an alternative.

Third, the reference is used in environments in which presenting DNAR as preferable is, itself, dispreferred. Reported speech is used to legitimate the identification of an option (DNAR), whose formulation may be problematic outside of this “reported” context. Mentioning evidence when making statements treats the matter as potentially being subject to upcoming disagreement. This can help to mitigate the potential disagreement, as well as to reduce perceived preference in regard to what is presented as best option [49]. Determining when DNAR is relevant is presented as something over which people/patients have authority, and not as something which might be medically indicated (over which the physician might have authority). The problematic nature of talking about relevance of DNAR is also displayed throughout the conversation: lengthy introductions, not listing options clearly to begin with (Excerpt 1), use of hesitations, descriptions depicting CPR as something detrimental and difficult to achieve, without benefit for the patient (Excerpt 2).

In particular, the reference to other patients’ decisions occurs after the initial part of the conversation, after the patients have already been given a chance to issue their preferences. In this way, it is employed to deal with some kind of “problem” or unsatisfaction with the patient’s preference. In the first excerpt, this problem resides in the fact that the patient is reluctant to express his preference despite several occasions. The reference is employed to clarify what the options are and to back up an option in particular. In the second excerpt, the problem is linked to the patient’s preference itself; reference to other people’s decisions displays that the physician treats the patient's response as inadequate because it might be against expectations or norms (what the physician thinks to be in the interest of the patient).

### Ethical implications of mentioning other people’s decisions in decision-making conversations

Patient decisions are influenced by how information is framed, how choices are listed and described [53, 54]. In regard to explanations, people can be strongly influenced by cues about what other people do [16]. Such references identify community or group norms and common sense, which exert a subtle but nonetheless existant pressure on patient choices. In virtue of its persuasive function [55] such reference has been uncontestably identified as a “nudge”: a way to influence someone’s behavior through how options are presented.

When it comes to healthcare communication, there is a long-standing divide between those who stand against use of nudges and those in (relative) favor of them. Those opposed argue that nudges, including those which encourage social comparison, may alter patients’ reasoning, reduce their voluntariness and threaten full patient autonomy [17, 56]. In contrast, there are others that consider nudges to be potentially ethically acceptable, as they aim for decisions that have the patient’s well-being in mind, as long as they are not misrepresentations (especially important in the case of reference to norms and social comparison), and that the power differentials between the messenger and the person nudged are not too great [16, 57, 58]. As Pecanac and Yankee show [48], physicians themselves are ambivalent towards considering the valence of this particular nudge (referring to what other patients say) as paternalistic or exemplary.

In our data, physicians employ reference to other patients’ decisions to refer to DNAR and to project it as desirable and/or relevant for the patient. Patients in both excerpts don’t deliver their responses or expected responses at the first occasions offered, though they select DNAR as final option. There is no way of telling with absolute certainty that this was due to the particular way in which options are presented by the physician, yet the use of social comparison warrants a reflection on its ethical implications.

Through the use of direct reported speech and by anchoring decisions in group membership, the information is presented as something that can be accepted at face value by the patient. As mentioned, in the first year of the study, hospital statistics showed that for 43% of the admitted patients CPR was documented as relevant. This means that none of the code statuses is significantly more representative for the population of patients admitted to this service (at least not without considering other socio-demographic or medical aspects). Presenting this information as something that patients “often” say, as in Excerpt 2, is an exaggeration of the reality. The fact that this information comes after the patient has offered a clear response strengthens its nudging character towards a decision that the physician seems to consider as more adequate. Compared to Excerpt 2, Excerpt 1 shows a less forceful use of social comparison, since there is no reference to the distribution of the decision within the population and since two options are presented (what “people” say is counterbalanced by what “other people” say). Rather, in Excerpt 1, the strength of the social comparison resides in the association of the decision against CPR with having lived a good live, which projects it as preferable. From an ethical point of view, reference to other people’s opinions might have an impact on autonomous deliberation in certain contexts, since providing patients with a decisional shortcut that is not based on personal prognosis but on membership issues.

The detailed analysis of its occurrence also informs us that physicians employ social comparison only when their other attempts at securing a decision (and even, in Excerpt 2, a “correct” decision), have failed. This seems to rejoin one of the central hypotheses of Festinger, one of the pillars of Social Comparison Theory, who sustained that people resort to using social comparison information only when objective information is not available [59, 60]. In Excerpt 1, CPR is first presented as a technique difficult to accomplish and strenuous in terms of activities and trajectory (going to intensive care). In Excerpt 2, the physician first refers to the patient’s personal prognosis in a way that displays a clear counter-indication towards CPR. Reference to what other people decide is only employed after other strategies for securing a response have been tried. As such, beyond advising physicians to avoid using social comparison unless it is rigorously applied (based on actual distribution of choices within the patient population), we equally want to draw attention to the need of discussing how physicians can approach this sensitive topic in a way that is humane and ethical, especially in situations in which CPR might not be medically indicated.

In this sense, one thing worth noting is that the context in which these discussions are led is perhaps not the most conducive for their finality. As we argue in our previous works [5, 6], admission interviews are primarily concerned with obtaining information from the patient (medical history, physical and cognitive exams). It is of no surprise that code status is addressed as an information that patients have and give to the physician, when in reality, this is by no means a unidirectional moment but a decisional one, in which choices should be made or reassessed, with the participation of both the physician and the patient. This means that instead of using information-oriented questions such as “what do you wish that we do?” (that allows for a vague answer and presupposes that patients have access to certain knowledge about potential choices) and “would you wish that we try to make it work?” (that only lists one option) physicians might consider other ways of engaging patients into talking about this topic. One alternative is the approach used in Advance Care Planning, consisting of first probing patient’s attitudes about the future and their health state, their understanding of quality of life as well as their fears and expectations, exploring their prior experience with planning and decision making, before discussing specific treatment preferences [61]. In this way, physicians would have an opportunity to anchor the information that they give about CPR (and other life-sustaining interventions) into the patient’s shared reflections about what is important to them and what is not. Not only would this be a way of circumventing the use of nudges, but it would facilitate physicians’ job of talking about the prospect of death in an environment oriented towards patients’ rehabilitation, and engage with patients whose CPR prognostic is unfavorable.

## Conclusion

One way that communication research can contribute to a better integration of ethics into decision-making communication is by identifying and investigating practices with a nudging potential. Our findings show that when discussing CPR, physicians may refer to decisions made by other people, especially when faced with a lack of decision making or with decisions incongruent with the medical evaluation. While this is essentially a way of talking about options, it can also convey a strong preference or desirability of DNAR orders when there is a problem with the patient’s initial preference—either because the patient couldn’t express one or because their decision is not compatible with what is presented as being in the patient’s best medical interest. Framing a decision as something someone else made calls out to a sense of identity that may lead patients to embrace norms that are associated with a desirable group. While this might facilitate the act of selecting a specific therapeutic option, it would do so following a sense of membership to a social category, that is not related to clinical criteria that should guide decision making.

The occurrence of this phenomenon also shows that talk about DNAR is a complex task for physicians. Referring to DNAR decisions as made by other patients is one way in which physicians might introduce DNAR without directly addressing the possibility of allowing for natural death. As other studies have showed, even in specialized healthcare contexts such as palliative care, bringing up end-of-life issues is challenging, even for experienced physicians, and often more subtle cues seem to be preferable to overt ones [33]. Our own findings on how physicians elicit patients’ preferences about CPR showed that patients’ choices for DNAR are often made in anticipation of an actual request (when given an opportunity) [6]. This supports the reflection that initiating open talk about end-of-life and, implicitly the relevancy of DNAR, might be confrontational for physicians and is best achieved through more subtle resources, such as references to DNAR being what other patients opt for.

## Supplementary Information

**Additional file 1**. **Appendix 1**: Transcription key adapted from the Jefferson (2004) transcription system.

**Additional file 2.** **Appendix 2**: Transcript in original language and translation for Conversation 35: Physician #14 (PHY14); Patient #46 (PAT46).

**Additional file 3. Appendix 3**: Transcript in original language and translation of Conversation 44 (Physician #15: PHY15; Patient #55: PAT55).

## Data Availability

The dataset supporting the conclusions of this article can be obtained from the main author upon reasonable request.
