# Supplementary material for: Decision-making ethics in regards to life-sustaining interventions: when physicians refer to what other patients decide
Source: BMC Med Ethics. 2022 Sep 2;23:91. doi: 10.1186/s12910-022-00828-2 (PMC9440599; doi:10.1186/s12910-022-00828-2)
Supplement: Supplementary file 1 — Additional file 1. Appendix 1: Transcription key adapted from the Jefferson (2004) transcription system. [file 12910_2022_828_MOESM1_ESM.pdf]

**Appendix 1. Transcription key adapted from the Jefferson (2004) transcription system**

| | |
|---|---|
| = | no discernable break between the turns/latching conversation |
| [ | point of overlap onset |
| ] | point of overlap end |
| (3.4) | length of silence, measured in seconds and tenths of seconds |
| (.) | micro pause (less than 0.2 of a second) |
| : | lengthening or stretching of the sound |
| - | cut-off or self-interruption |
| . | falling intonation |
| , | continuing intonation |
| ? | talk ending with rising intonation |
| h | hearable outbreath |
| ·h | hearable inbreath |
| underline | emphasized talk |
| °no no° | talk is quieter than surrounding talk |
| >no no< | talk is faster than surrounding talk |
| ((word)) | transcriber's description |
