# Supplementary material for: Decision-making ethics in regards to life-sustaining interventions: when physicians refer to what other patients decide
Source: BMC Med Ethics. 2022 Sep 2;23:91. doi: 10.1186/s12910-022-00828-2 (PMC9440599; doi:10.1186/s12910-022-00828-2)
Supplement: Supplementary file 2 — Additional file 2. Appendix 2: Transcript in original language and translation for Conversation 35: Physician #14 (PHY14); Patient #46 (PAT46). [file 12910_2022_828_MOESM2_ESM.pdf]

Appendix 2 : Transcript in original language and translation for Conversation 35 : Physician #14 (PHY14); Patient #46 (PAT46)

X1 PHY14: notre objectif ici est de vous apprendre, comme vous dites,
our objective here is to you teach, as you say,
our objective here is to teach you, as you say,

X2 dans la gestion quotidienne, finalement à apprendre à faire juste avec
in the management daily, ultimately to teach to do right with
in dealing with daily life, ultimately teaching you to do make do with

X3 cette nouvelle jambe, enfin voilà. •hh (1.5) oké. ts
this new leg, finally there. •hh (1.5) okay. ts
the new leg, well that's it. okay. ts

01 on vous pose toujours cette question quand vous arrivez à l'hôpital,
one you put always this question when you come to the hospital,
we always ask you this question when you arrive at the hospital,

02 (0.4) ts euh:: je vous la repose parce qu'on doit toujours la
(0.4) ts euh:: I you it re-ask because that one have always it
(0.4) ts uh:: I am re-asking it to you because we always have

03 reposer à tous les patients qui arrivent ici, •hh c'est
re-ask to all the patients who arrive here, •hh it's
to re-ask it to all patients who arrive here, •hh it's

04 >qu'est-ce que vous souhaitez qu'on fasse< si jamais votre cœur s'arrête?
>what would that you wish that one does if ever your heart itself stops?
>what would you wish that we do< if ever your heart stops?

05 (1.1)

06 PHY14: euh: (0.5) dans le sens que: (0.2) voilà c'est: (0.7) la réanimation
uh: (0.5) in the sense that: (0.2) there it's: (0.7) the resuscitation
uh: (0.5) in the sense that: (0.2) well it's: (0.7) resuscitation

07 c'est: (2.9) >c'est quand même quelque chose qui est pas, qui n'est pas
it's: (2.9) >it's when though some thing that is not, that not is not
it's: (2.9) >it's actually something that is not, that is not

08 magique=y suffit pas de represser sur le bouton=de represser sur le
magic=it sufficient not to re-press on the button=to repress on the
magic=it's not enough to re-press the button=to repress on the

09 cœur il repart et tout va bien.< j'entends c'est: c'est quelque chose
heart it restarts and all go well.< I hear it's: it's some thing
heart it restarts and all is well.< I mean it's: it's something that

10 de toute façon euh les chances de réussir une >réanimation sont de toute
of all way uh the chances to succeed a >resuscitation are of all way
in any case uh the chances to succeed with a >resuscitation are in any

11 façon très faibles.<
case very weak.<

12 PAT46 : °(mh mh)°

13 PHY14: •h avec de toute façon si on fait une réanimation
•h with of all way if one does a resuscitation
•h with in any case if we do a resuscitation

14 après un passage >il faut aller au ((hôpital)) il faut les soins
after a passage >one has to go to ((hospital)) one needs the care
afterwards go through >one has to go to ((hospital)) one needs intensive

15 intensifs=enfin j'entends voilà ça veut dire beaucoup de CHOSES?<
intensive=finally I hear there it wants say many of THINGS?<
care=well I mean that's it means a lot of THINGS?<

16 (1.0)

17 PHY14: je pose cette question simplement parce
I ask this question simply because
I ask this question simply because

18 qu'il y a des gens pour qui <c'est très clair que> ils disent >non mais
that it there are people for whom <it's very clear that> they say >no but
→ **there are people for whom <it's very clear that> they say >no but**

19 écoutez si ça s'arrête, moi j'ai bien< vécu: je veux plus rien
listen if that it stops, me I've well< lived: I want more nothing
→ **listen if it stops, myself I've lived< well: I don't want to do: anything**

20 fai:re. (1.0) et il y en a d'autres qui voilà ils disent non mais moi
do:. (1.0)and it there are others that there they say no but I
→ **else. (1.0) and there are others who well they say no but myself**

21 c'est quand même important qu'on- qu'on fasse quelque chose. enfin voilà.
it's when though important that one- one does some thing. finally there.

	→	**it’s actually important that we- that we do something. well that’s it.**
22		(1.3)
23	PAT46:	non mais disons il faut- ça sert à rien de nous réanimer si
		no but say it needs- this uses to nothing to us reanimate if
		**no but let’s say one has to- it’s no use to resuscitate us if**
24		il y a des séquelles très graves quand même après. (0.3)
		it there are repercussions very serious when though afterwards. (0.3)
		**anyway there are very serious repercussions afterwards. (0.3)**
25		que quelque chose soit pas bien.=
		that some thing being not well.=
		**that something might not be well =**
26	PHY14:	=alors ça le problème c’est qu’on ne sait jamais à l’avance.=
		=so that the problem it’s that one not know ever in the advance.=
		**=so this the problem is that we never know in advance.=**
27	PAT46:	=°ouais°=
		**=°yeah°=**
28	PHY14:	=on ne peut pas se dire, on regarde la personne et puis on dit
		=one not can not oneself tell, one looks the person and then one says
		**=we can’t tell ourselves, we look at the person and then we say so**
29		alors elle elle aura des séquelles et puis elle
		then she she will have repercussions and then she
		**she she will have repercussions and then she**
30		elle n’en aura pas. ·hh c’est ça le problème.
		she not it have not. ·hh it’s that the problem.
		**she won’t have. ·hh that’s the problem.**
31		(1.2)
32	PAT46:	non [mais:
		**no [but:**
33	PHY14:	[effectivement si-
		**[indeed if-**
34	PAT46:	faut pas: réanimer.
		**should not: resuscitate.**
