# Supplementary material for: Decision-making ethics in regards to life-sustaining interventions: when physicians refer to what other patients decide
Source: BMC Med Ethics. 2022 Sep 2;23:91. doi: 10.1186/s12910-022-00828-2 (PMC9440599; doi:10.1186/s12910-022-00828-2)
Supplement: Supplementary file 3 — Additional file 3. Appendix 3: Transcript in original language and translation of Conversation 44 (Physician #15: PHY15; Patient #55: PAT55). [file 12910_2022_828_MOESM3_ESM.pdf]

Appendix 3 : Transcript in original language and translation of **Conversation 44 (Physician #15: PHY15; Patient #55: PAT55)**

01 PHY15: s'il y a quelque chose qui arrive à votre cœur, qu'il
if it there is something that happens to your heart, that it
if there is something that happens to your heart, that it
02 s'arrête, tout d'un coup, (0.4) ou que le poumon s'arrête, tout
itself stops, all of one beat, (0.4) or that the lung itself stops, all
stops, all of a sudden, (0.4) or that the lung stops, all
03 d'un coup de fonctionner, (0.7) c'qui est quand même une complication
of one beat to function, (0.7) that which is when though a complication
of the sudden from functioning, (0.7) which is really a se:rious,
04 gra:ve, (0.7) est-ce que vous aimeriez qu'on refasse (.) qu'on essaie
se:rious, (0.7) it it that you wish that one redo (.) that one try
complication (0.7)would you wish that we remake (.) that we try
05 de le refaire marcher, (0.6) faire un choc électrique, (0.4) mettre un
to it redo work, (0.6) do a choc electric, (0.4) put a
to remake it work, (0.6) do an electric shock, (0.4) put a
06 tube éventuellement pour soutenir le poumon?
tube eventually for sustaining the lung?
tube maybe to sustain the lung?
07 (0.4)
08 PAT55: ouais, j'crois que oui alors.
yeah, I think that yes so.
yeah, so I think that yes.
09 PHY15: ·h parce que (.) c'est vrai qu'à votre âge, vous avez euh nonante-trois
·h because (.) it's true that at your age, you are uh ninety-three
10 ans hein?
years old huh?
11 PAT55: nonante-quatre.
ninety-four.
12 PHY15: vous avez nonante-quatre, vous avez aussi pas mal de maladies: donc
you are ninety-four, you have also not bad of illnesses: so
you are ninety-four, you also have quite a lot of illnesses: so
13 c'est aussi la réanimation comme on [la fait]
it's also the resuscitation as we [do it]
14 PAT55: [c'est ça ouais,] ouais.
[that's it yeah,] yeah.
15 PHY15: c'est pas une chose ano- euh anodine hein? c'est pas quelque chose qui:
it's not a thing har- uh harmless huh? it's not some thing that:
it's not a harm- harmless thing huh? it's not something that:
16 qui est sans complication.
that is without complication.
17 (0.3)
18 PAT55: voilà.
right.
19 (0.4)
20 PHY15: donc, (.) on ne peut pas prévoir les complications quand on essaie de:
so, (.) one not can not foresee the complications when one tries to:
so, (.) we cannot foresee the complications when we try to:
21 d'intervenir comme ça.
to intervene linke that.
to intervene like this.
22 (0.4)
23 PAT55: ouais.
yeah.
24 (0.4)
25 PHY15: c'est ce qu'on appelle,
it's that that one calls,
it's what we call,
26 souvent les patients ils nous disent pas d'acharnement.
often the patients they us tell no of futility.
→ **often the patients they tell us no futile care.**
27 (0.4)
28 PAT55: ah ouais c'est ça.
oh yeah that's it.
29 PHY15: vous partagez cet avis?
you share this opinion?

30	PAT55:	je trouve c’est bien hein, pas d’acharnement.<br>**I find it’s good huh, no futility.**
31	PHY15:	pas d’acharnement.<br>**no futility.**
32	PAT55:	ouais,<br>**yeah.**
33	PHY15:	vous ne voulez pas donc.<br>*you not want not so.*<br>**so you don’t want.**
34	PAT55:	non.<br>**no.**
35	PHY15:	et si: il fallait vous transférer au ((hôpital)) pour une<br>**and if: we had to transfer you to the ((hospital)) for an**
36		maladie aigue, vous seriez d’accord?<br>**acute disease, you would agree?**
